# Accuracy of Death Certificates for Children: A Population-Based Retrospective Analysis

**DOI:** 10.3390/pediatric17060115

**Published:** 2025-11-03

**Authors:** Masahito Yamamoto, Masahito Hitosugi, Eisuke Ito, Kohei Takashima, Mami Nakamura, Seiro Narumiya, Yoshihiro Maruo

**Affiliations:** 1 Department of Pediatrics, Nagahama Red Cross Hospital, Nagahama 526-8585, Shiga, Japan; masahito.yamamoto@me.com (M.Y.); hikochu18@hotmail.com (S.N.); 2 Department of Legal Medicine, Shiga University of Medical Science, Otsu 520-2192, Shiga, Japan; mamin@belle.shiga-med.ac.jp; 3 Department of Pediatrics, Saiseikai Shiga Hospital, Ritto 520-3046, Shiga, Japan; eisuke@saiseikai-shiga.jp; 4 Department of Pediatrics, Shiga University of Medical Science, Otsu 520-2192, Shiga, Japan; takachan@belle.shiga-med.ac.jp (K.T.); maruo@belle.shiga-med.ac.jp (Y.M.)

**Keywords:** death certificate, child death review, cause of death, accuracy

## Abstract

**Background/Objective**: Accurate determination and documentation of causes of death in children are essential for generating reliable mortality statistics and guiding public health strategies. Previous studies have reported frequent inaccuracies in pediatric death certificates (DCs), including the use of vague terms, omissions of relevant conditions, and variability across physician specialties. This study evaluated the accuracy of pediatric DCs in Shiga Prefecture, Japan; identified common errors in these DCs; and examined changes in the underlying causes of pediatric death before and after the COVID-19 pandemic. **Methods**: We performed a population-based retrospective review of 391 DCs for individuals under 18 years issued between 2015 and 2023. Two pediatricians and two forensic pathologists independently reviewed each DC, assessed accuracy, and classified errors using predefined criteria. Error rates were compared by physician specialty. Underlying causes of death were reassessed into ten categories, and their distributions were compared between 2015–2019 and 2020–2023. **Results**: Overall, 30.9% of DCs contained errors. The error rates differed by physician specialty: obstetricians had the highest error rate (92.9%), whereas forensic physicians had the lowest (8.4%). The most common error type was the use of non-specific mechanisms such as “cardiac arrest” or “respiratory failure”, rather than the actual causes of death. Congenital anomalies were often listed under other significant conditions contributing to death and not as an underlying cause of death. After the onset of the COVID-19 pandemic, deaths from acute diseases declined from 16.8% to 4.0%, while deaths from congenital disorders increased from 12.6% to 24.3%. **Conclusions**: Pediatric DCs often contain errors, particularly those completed by obstetricians. Misclassifying mechanisms as causes of death and underreporting congenital anomalies remain the main challenges. Strengthening physician education and introducing systematic review processes are essential to improve accuracy, clarify regional mortality trends, and guide effective public health interventions.

## 1. Introduction

According to the Global Burden of Diseases, Injuries, and Risk Factors Study, the annual number of live births worldwide peaked at 142 million in 2016 but declined to 129 million by 2021. Global fertility rates have continued to decline and are projected to reach a total fertility rate of 1.83 by 2050 and 1.59 by 2100 [1]. In response to these demographic shifts, the United Nations adopted the Sustainable Development Goals to promote healthy lives and well-being for all children [2]. Accurate documentation of live births, stillbirths, and deaths serves as a fundamental indicator of child health at both the regional and national levels. In particular, cause-specific mortality data are essential for guiding public health policies and interventions aimed at improving child health outcomes. A death certificate (DC) is a legal and medical document that formally records an individual’s death. Part 1 of the DC outlines the sequence of medical conditions leading directly to death, with the underlying cause determined according to the International Classification of Diseases (ICD). Part 2 includes other significant conditions that contributed to death but were not part of the causal sequence. Accurate identification of the underlying cause of death is critical for generating reliable mortality statistics and for informing health system strategies and policy development. However, multiple studies have shown that misclassification and inaccuracies in all-cause mortality reporting are common in hospital-issued DCs.

Previous research has highlighted deficiencies in vital registration systems, including poor-quality cause-of-death data [3,4,5,6,7,8,9,10,11]. Several studies have specifically assessed the accuracy of pediatric DCs. A quality assurance survey conducted at 20 hospitals in Ghana reported that only 36.5% of pediatric DCs recorded an accurate cause of death when cross-referenced with discharge records [4]. In pediatric hospitals in Sudan, only 1.8% of DCs were found to be fully and correctly completed [7]. In a population-based study conducted in Japan, Urabe et al. examined DCs for individuals under 20 years of age [12] and reported a low autopsy rate of 13.5%, with discrepancies found between the DCs and autopsy findings in 37.7% of cases. Notably, 70% of sudden infant death syndrome cases were diagnosed without an autopsy confirmation [12]. A prior study reviewed pediatric DCs issued in Shiga Prefecture, Japan [13], and found vague descriptions such as “unknown cause of death” or “under inspection.” In Japan, medical autopsy is performed for diseased death at the hospital. Because the cause of death was already diagnosed by the clinicians, pathologists do not fill the death certificates. In our series, if the cause of death was not determined clinically or by postmortem external investigations, forensic autopsy was performed and cause of death was determined. If the autopsy was planned immediately after death (suspecting homicide, deep death investigation is required without witness, and so on), forensic pathologist first handle the body and filled the death certificate. When the pediatrician or general physician externally examined the body and could not determine the cause of death, they fill the death certificate as “Unknown” or “Under inspection”, and then, forensic autopsy was performed. In some cases, the information from forensic autopsies helped to clarify these ambiguities, underscoring the utility of integrating autopsy findings with DC data. Assessing the causes of death at the regional level can enable the implementation of targeted public health interventions to reduce preventable mortality and strengthen healthcare infrastructure. These efforts are contingent upon improving the quality of DCs. Despite existing studies, no prior population-based research has comprehensively evaluated the adequacy of death certificate descriptions for individuals under 18 years of age in Japan.

The present study aimed (1) to assess the accuracy of pediatric DCs and identify the most common types of errors and (2) to propose effective strategies for improving the quality of death certification for children.

## 2. Materials and Methods

This population-based retrospective study reviewed all DCs submitted to Shiga Prefecture, Japan, between 2015 and 2023 for individuals under 18 years of age. Shiga Prefecture, located in central Japan, spans an area of 4017 km^2^ and had a population of 1,406,103 in 2023, including 9249 live births. The study was conducted with formal approval from the Ministry of Health, Labour and Welfare of Japan. Two certified pediatricians and two certified forensic pathologists independently evaluated each DC to identify and classify errors. The reviewers assessed the language and structure of the recorded information to determine whether the entries were reasonable and aligned with accepted standards [14]. The evaluation was performed blindly without the certifier’s name, specialty, and affiliation being shown. Following individual reviews, all findings were discussed to reach a consensus on the classification of errors, which were categorized according to predefined criteria (Table 1). The discussion resulted in no disagreements with respect to the classification of errors. Regarding Group C, for example, when a patient died of congenital heart disease due to trisomy 18, but trisomy 18 was not listed as the underlying cause or was listed under other significant conditions contributing to death (Part 2). We defined the major error as relating to the cause of death. Other mistakes included miswriting the interval between onset and death, not marking the “place of death”, not including additional information on the external cause of death, and not writing the time of death in the 12-h time format, were defined as minor error. In cases where the original DCs listed the cause of death as “Unknown” or “Under inspection,” updated versions of the DCs, reflecting findings from subsequent forensic autopsy, were reviewed. It is important to note that the purpose of this study was not to validate the diagnostic accuracy of the recorded causes of death; therefore, the original medical records were not examined.

The certifying physician’s specialty, as listed on each DC, was classified into one of four groups: pediatricians (including pediatric surgeons), physicians and surgeons who primarily treat adults, obstetricians, and forensic physicians. Forensic physicians include forensic pathologists who perform forensic autopsies and clinicians with forensic training who determine the cause of death for unnatural deaths.

Based on a comprehensive review of all diagnostic descriptions included in each DC, the research team reclassified the underlying cause of death where necessary, regardless of the originally documented cause. The revised causes were then grouped into the following categories based on previous studies [15]: homicide, suicide, unintentional injury, malignant disease, chronic disease (excluding malignancies), infectious disease, acute disease (excluding infections), congenital disease, perinatal disease, and sudden infant death syndrome (SIDS)/unknown. The distribution of these classifications was compared across two time periods: before (2015–2019) and after (2020–2023) the onset of the COVID-19 pandemic.

Categorical variables were summarized as frequencies and percentages. Because the age distribution of the decedents was non-normal, age was reported as median with interquartile range. Comparisons of error prevalence across physician specialties and error types were performed using Fisher’s exact tests. Post hoc residual analysis was conducted to identify significant deviations from expected frequencies, with adjusted residuals greater than 1.96 considered statistically significant. For each category, the primary effect measure was the risk difference (RD) (post−pre) with Newcombe 95% CI; two-sided Fisher’s exact tests were used to calculate the *p*-values. Multiple comparisons were handled via the Benjamini–Hochberg false discovery rate (per table). All tests were two-sided (*α* = 0.05); the results are presented as n/N (%), RD in percentage points, *p*-values, and *q*-values. All statistical analyses were conducted using EZR (Saitama Medical Center, Jichi Medical University, Saitama, Japan), a graphical user interface for R (The R Foundation for Statistical Computing, Vienna, Austria).

This study was approved by the institutional ethics committee (approval no. R2020-164).

## 3. Results

### 3.1. General Background

**Figure 1:**
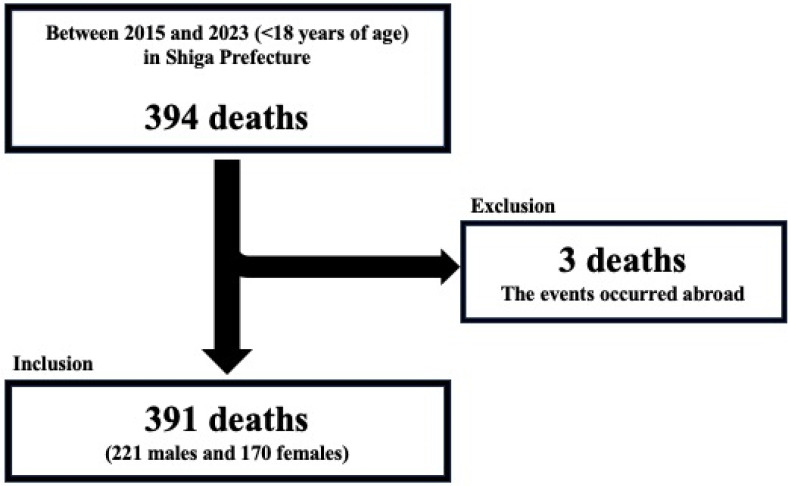
Research participants.

**Figure 2:**
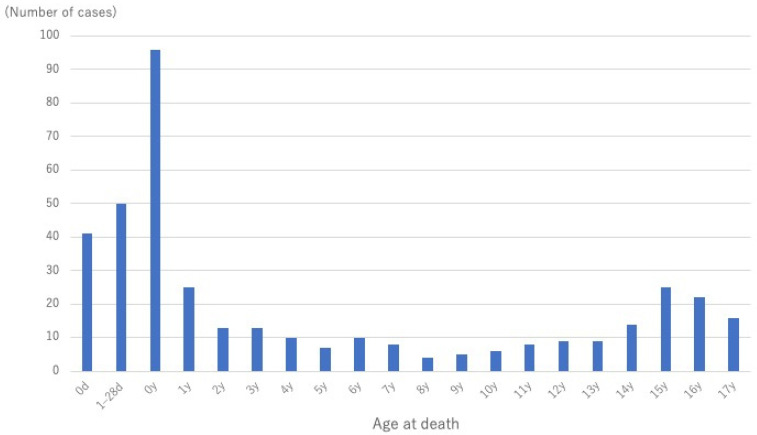
Age-distribution of all decedents (n = 391) included in this study.

During the study period, a total of 394 deaths were recorded. Of these, 391 cases (221 males and 170 females) were included in the analysis, excluding 3 deaths for which the cause could not be confirmed because the events occurred abroad (Figure 1). The median age of the decedents was 1 year (interquartile range: 0–11 years). The age distribution of all cases is illustrated in Figure 2.

Regarding the specialty of the certifying physician, pediatricians accounted for the majority of DCs (226 cases), followed by forensic physicians (83 cases), physicians and surgeons who treat adult patients (61 cases), and obstetricians (14 cases). A total of 104 cases (26.6%) underwent autopsy. Of these, 78 were forensic autopsies and 26 were medical autopsies. Among them, errors were identified in the DCs of 15 cases, with the following breakdown: 8 cases corresponded to error A, 0 case of error B, 5 cases of error C, and 4 cases of error D.

### 3.2. Prevalence of Errors

Among the 391 reviewed DCs, 121 (30.9%) were determined to contain inappropriate or inadequate entries. When stratified by physician specialty, the prevalence of errors was highest among obstetricians at 92.9% (13 of 14 cases), followed by pediatricians at 34.1% (77 of 226 cases), physicians and surgeons at 32.8% (20 of 61 cases), and lowest among forensic physicians at 8.4% (7 of 83 cases) (Table 2). The error rate was significantly higher for obstetricians and significantly lower for forensic physicians compared with other specialties (*p* < 0.01).

In terms of error classification, “Group A: Inappropriate cause of death” was the most frequently observed category, accounting for 82 of the 121 error cases (67.8%). Within this group, the most common subcategory was “A3: Inappropriate injury or disease name,” representing 39.7% of Group A errors (Table 3). No statistically significant differences in error distribution were observed across physician specialties for Groups A to D. Similarly, no significant differences were found among the subcategories of A1 to A4 within Group A across physician specialties. Notably, for errors attributed to obstetricians, Group A errors were predominant (92.3%), with “A1: heart failure or respiratory failure as end-stage condition listed under immediate cause of death” being the most frequent type of errors.

### 3.3. Comparison of Distribution of Underlying Causes of Death

The distribution of the revised underlying causes of death was compared between the pre-pandemic (2015–2019) and pandemic (2020–2023) periods (Table 4). The proportion of deaths attributed to acute diseases declined significantly from 16.8% (36 cases) to 4.0% (7 cases) (*p* < 0.01). In contrast, the proportion of deaths resulting from congenital disorders increased significantly from 12.6% (27 cases) to 24.3% (43 cases) (*p* < 0.01). Among infants, acute causes decreased significantly (RD−10.8%, 95% CI −19.3% to −0.3%, *p* = 0.005, *q* = 0.007), whereas congenital causes increased significantly (RD + 18.9%, 95% CI + 0.3% to +36.4%, *p* = 0.005, *q* = 0.007). Among children/adolescents, acute causes also decreased significantly (RD −15.6%, 95% CI−27.6% to −2.2%, *p* = 0.002, *q* = 0.007), but congenital causes showed a nonsignificant increase (RD +7.5%, 95% CI−3.4% to +18.2%, *p* = 0.074, *q* = 0.074). Although there were two COVID-19-related deaths recorded in 2022, the overall prevalence of deaths linked to infectious diseases showed a nonsignificant increase from 5.1% (11 cases) to 7.9% (14 cases). Additionally, the proportion of deaths attributed to suicide demonstrated a slight increase, although this change did not reach statistical significance.

## 4. Discussion

Khelil et al. conducted a comprehensive review of the literature on errors in death certification and reported that the proportion of DCs completed without any error ranged widely from 0% to 80% [5]. In the present study, 30.9% of pediatric DCs were found to contain inadequate descriptions. This rate is comparatively lower than those reported in several previous investigations [3,5,6,8,9,10,11]. One possible explanation for this relatively improved performance is the influence of regulatory and educational initiatives in Japan. The Basic Act for Promoting the Determination of Cause of Death, which came into effect in April 2020, emphasizes the importance of accurate cause-of-death documentation. Preparatory measures aligned with this Act were initiated as early as 2015, including the establishment of a regional council for promoting the determination of causes of death. As part of these efforts, forensic autopsies were recommended for cases of sudden unexpected death, physicians were advised not to certify causes of death without sufficient evidence, and annual seminars on correct DC completion were conducted through local medical associations. These interventions may have contributed to the improved quality of death certification observed in this study. 

Over the nine-year study period, an increasing trend in the prevalence of deaths attributed to congenital anomalies was observed. This finding is consistent with global data indicating a rising contribution of congenital abnormalities to early mortality, particularly as deaths from other causes decline [16]. The impact of the COVID-19 pandemic may have further amplified this trend. In the study region, the perinatal care system is typically organized into four regions, allowing for localized, region-specific care. However, during the pandemic, this structure was temporarily dismantled, and hospitals were designated as either COVID-19 referral centers or non-COVID facilities. As a result, some pregnant women might have been unable to access timely or adequate perinatal services, as they were required to seek care outside their designated region. This disruption could have contributed to an increased risk of adverse perinatal outcomes. In Europe, congenital anomalies were estimated to account for 26% of infant deaths, 16% of deaths in children aged 1 to 4 years, and 9% of deaths in those aged 5 to 9 years between 2000 and 2015 [17]. The present finding that 24.3% of deaths from 2020 to 2023 were attributed to congenital disorders is consistent with these estimates. However, this trend may shift as healthcare systems recover from the pandemic, warranting ongoing monitoring of cause-of-death distribution in the pediatric population. 

In this study, the underlying cause of death was reassessed by reviewing all descriptions recorded in the DCs. In several cases, specific congenital conditions such as Down syndrome, trisomy 13 (T13), and trisomy 18 (T18) were listed in Part 2 of the DC but were absent from Part 1, which should have outlined the causal sequence leading directly to death. According to ICD guidelines, the underlying cause of death refers to the disease or injury that initiated the chain of events ultimately resulting in death and serves as the foundation for compiling mortality statistics. A previous analysis of cases involving T13 and T18 [18] reported that in 5 out of 12 cases, these diagnoses were not identified as the underlying cause of death, primarily as a result of unclear or inaccurate instructions in the cause-of-death section. Data from 13 European congenital anomaly registries further support these findings, showing that in 30% of infant deaths involving major congenital anomalies, the underlying cause of death was not recorded as such [19]. Enhancing the accuracy and completeness of DCs, particularly in recording congenital anomalies, is therefore a critical challenge that must be addressed to advance both medical practice and public health policy.

Our findings indicate that non-specific mechanisms of death, such as cardiac failure, respiratory failure, and cardiac arrest, were cited as the cause of death in 25.6% of the reviewed cases. This type of error, which conflates physiological mechanisms with specific etiologies, has been similarly documented in previous studies. For instance, such errors were reported in 21.0% of DCs issued by an emergency department in Tunisia [5] and in 26.5% of those issued by a tertiary care hospital in India [9]. These inaccuracies typically arise from confusion between the mechanism of death, a non-specific physiological or biochemical derangement contributing to death, and the actual cause of death, which refers to a well-defined pathological process or diagnosis. In Greece, correctly completed DCs accounted for only 39.4% of the reviewed cases, with the most frequent error (34.5%) being the substitution of the mechanism of injury for the actual cause of death [11]. A more detailed analysis conducted by the authors of the Tunisian emergency department study found multiple issues contributing to the inaccuracy of DCs [5], including omission of the cause-of-death sequence in 38.2% of cases, improper sequencing in 31.2%, unacceptable causes of death in 21%, and incorrect inclusion of mechanisms of death in 20% [5]. These findings are in close alignment with our results and highlight a pervasive lack of awareness regarding appropriate death certification practices. Specifically, they underscore the need for physicians to understand both ICD coding conventions and how to identify the most likely pathological event that initiated the fatal sequence.

With regard to physician specialty, our study demonstrated significant differences in the frequency and types of errors. Obstetricians had the highest rate of inadequate death certification at 92.9%, while forensic physicians exhibited the lowest rate at 8.6%. Error classification revealed that Group A errors—those related to inappropriate causes of death—were predominant among obstetricians, whereas Group D errors were more commonly made by forensic physicians, which was considered a minor and not a major concern. Notably, 26.0% of the deaths in our cohort occurred within the first day of life, and in these cases, obstetricians were typically responsible for certifying the death. A unique and important observation from this study was the tendency among obstetricians to document maternal complications, such as shoulder dystocia, as the cause of death on the infant’s DC. This practice reflects a fundamental misunderstanding of the distinction between maternal and neonatal mortality causation and represents a novel finding that has not been highlighted in the previous literature. Recognizing this pattern provides an opportunity to refine certification practices for neonatal deaths and improve the accuracy and utility of mortality statistics, particularly for infants who die shortly after birth. In addition to pediatricians, obstetricians are often responsible for certifying neonatal deaths. Therefore, the educational curriculum for certified obstetricians should incorporate training not only in neonatal resuscitation but also in the appropriate completion of DCs for neonates who could not be resuscitated and subsequently died. In the future, comprehensive surveys among obstetricians regarding their experience in filling DCs should be conducted while ensuring educational opportunities for those with less experience. The low error rate observed among forensic physicians is likely attributable to their extensive experience in postmortem investigations and the high volume of DCs they complete, supported by their specialized training in death certification practices.

Given the pediatric focus of the present study, it was initially anticipated that pediatricians would demonstrate a lower rate of death certification errors compared with physicians or surgeons who treat adult populations. However, no statistically significant difference was observed between the two groups (34.1% vs. 32.8%). This finding is consistent with earlier reports indicating that pediatricians may, in fact, produce DCs with a higher degree of inaccuracy than other clinical specialists [20,21]. Other studies have noted that general practitioners tend to complete DCs with greater accuracy than specialists [6,22], potentially because of their broader familiarity with the patients and the continuity of care. These observations suggest that physician specialty alone does not fully explain the occurrence of errors; additional systemic and contextual factors are likely involved.

To improve the quality of death certification, several strategies should be implemented. First, DCs should be cross-checked by a senior clinician directly involved in the care of the deceased patient. Additionally, there must be a broader shift in physician awareness regarding the critical role of DCs in shaping epidemiological data, informing public health policy, and guiding medical research. As Murray et al. have emphasized, accurate identification of causes of death largely depends on the examiner’s training and experience, and the quality of certification is closely tied to the rigor of such training programs [23]. Repeated educational interventions and structured training modules have been shown to significantly improve DC accuracy [24,25]. In light of these findings and our study results, we strongly recommend the implementation of regular, postgraduate hands-on training sessions for all clinicians who are responsible for completing DCs. 

This study has several limitations. First, we did not validate the DC descriptions against the corresponding medical records. Although the written content of the DCs allowed for the reassessment of the underlying cause of death, incorporating data from the full medical record would likely enhance diagnostic accuracy. Future studies should aim to validate DC entries against comprehensive clinical data. Second, the reasons underlying erroneous or incomplete entries could not be ascertained. Understanding the specific misconceptions or knowledge gaps that contribute to misclassification may aid in designing more effective interventions. Providing individualized feedback to certifying physicians may also be a valuable strategy for quality improvement. Third, this study was conducted in a single Japanese prefecture where annual training seminars are held as part of a regional initiative to improve death certification. It is possible that different challenges may exist in other regions of Japan. Therefore, similar population-based studies should be undertaken nationwide to assess regional variability and inform broader national strategies. Fourth, as we predicted that the number of death certificates filled by obstetricians was small, we did not invite any obstetricians as reviewers for this study. However, following the present results, further analyses would be performed with the review team including obstetricians.

## 5. Conclusions

Errors are frequently observed in pediatric DCs, particularly those completed by obstetricians. Misclassification of cause of death and mechanism of death, along with underreporting of congenital anomalies, remain major challenges. Strengthening physician education on DC completion and implementing systematic review processes are essential for improving accuracy, clarifying regional mortality patterns, and guiding effective public health interventions.

## Figures and Tables

**Table 1:** Classification scheme for types of death certificate errors.

Group	Error Description
A	Inappropriate cause of death
A1	Heart failure or respiratory failure as end-stage condition listed under immediate cause of death
A2	The order of cause of death is wrong
A3	Inappropriate injury or disease name
A4	Cause of death listed as unknown without an autopsy
B	Inappropriate type for manner of death
C	Underlying cause of death was not listed or was listed under other significant conditions contributing death
D	Minor mistakes

**Table 2:** Prevalence of inadequately completed death certificates by physician specialty.

Specialty	Prevalence	Fisher’s *p*-Value (vs. Other Professionals)
Pediatrician	34.1% (77/226)	0.07
Physician and Surgeon	32.8% (20/61)	0.65
Forensic physician	8.4% (7/83)	<0.01
Obstetrician	92.9% (13/14)	<0.01
Total	30.9% (121/391)	−

**Table 3:** Distribution of error types in death certificates according to physician specialty.

Type of Error	Pediatrician	Physician and Surgeon	Forensic Physician	Obstetrician	Total
A	63.6%	80.0%	57.1%	92.3%	67.8%
A1	23.4%	25.0%	0.0%	61.5%	25.6%
A2	5.0%	10.0%	0.0%	7.7%	5.0%
A3	39.7%	60.0%	42.9%	23.1%	39.7%
A4	3.3%	0.0%	14.3%	7.7%	3.3%
B	4.1%	5.0%	0.0%	0.0%	4.1%
C	28.9%	15.0%	0.0%	23.1%	28.9%
D	17.4%	10.0%	42.9%	38.5%	17.4%

**Table 4:** Comparison of the revised underlying causes of death before and during the COVID-19 pandemic.

Manner of Death	Cause of Death	Period		RD (Post − Pre), pp	95% CI, pp	Fisher’s *p*-Value	BH *q*-Value
		2015–2019 (Pre)	2020–2023 (Post)				
Homicide		2 (0.9%)	0 (0%)	−0.90%	(−3.3%, 1.9%)	0.50	0.58
Suicide		13 (6.1%)	20 (11.3%)	5.20%	(−2.7%, 13.2%)	0.07	0.23
Accidental death		37 (17.3%)	34 (19.2%)	1.90%	(−8.8%, 12.8%)	0.69	0.69
Disease	Malignant Diseases	28 (13.1%)	14 (7.9%)	−5.20%	(−13.5%, 3.6%)	0.10	0.26
	Acute Diseases	36 (16.8%)	7 (4.0%)	−12.90%	(−20.5%, −4.5%)	<0.01	<0.01
	Chronic Diseases	9 (4.2%)	3 (1.7%)	−2.50%	(−7.2%, 2.6%)	0.24	0.40
	Congenital Diseases	27 (12.6%)	43 (24.3%)	11.70%	(0.8%, 22.3%)	<0.01	0.02
	Perinatal Diseases	40 (18.7%)	38 (21.5%)	2.80%	(−8.4%, 14.1%)	0.53	0.58
	Infectious Diseases	11 (5.1%)	14 (7.9%)	2.80%	(−4.2%, 9.9%)	0.30	0.43
Unknown/SIDS		11 (5.1%)	4 (2.3%)	−2.90%	(−8.1%, 2.8%)	0.19	0.38
Total		214 (100%)	177 (100%)	−	−	−	−

Notes. RD is expressed as percentage points (pp), calculated as post-proportion minus pre-proportion. 95% CIs calculated based on Newcombe’s method (Wilson score). Two-sided Fisher’s exact tests per category; Benjamini–Hochberg false discovery rate across the 10 categories, reported as q-values. Abbreviations: SIDS, sudden infant death syndrome; RD, risk difference; CI, confidence interval; BH, Benjamini–Hochberg.

## Data Availability

The original contributions presented in this study are included in the article. Further inquiries can be directed to the corresponding author.

## Abbreviations

The following abbreviations are used in this manuscript:

DC	Death certificate
ICD	International Classification of Diseases
SIDS	Sudden infant death syndrome
EZR	Easy R (graphical user interface for R)
COVID-19	Coronavirus disease 2019
RD	Risk difference
CI	Confidence interval
BH	Benjamini–Hochberg
